# Chemically treated plasma Aβ is a potential blood-based biomarker for screening cerebral amyloid deposition

**DOI:** 10.1186/s13195-017-0248-8

**Published:** 2017-03-22

**Authors:** Jong-Chan Park, Sun-Ho Han, Hyun Jin Cho, Min Soo Byun, Dahyun Yi, Young Min Choe, Seokjo Kang, Eun Sun Jung, Su Jin Won, Eun Hye Kim, Yu Kyeong Kim, Dong Young Lee, Inhee Mook-Jung

**Affiliations:** 1 Department of Biochemistry and Biomedical Sciences, Seoul National University, College of Medicine, 103 Daehak-ro, Jongno-gu, Seoul, 110-799 South Korea; 2 Neuroscience Research Institute, Seoul National University, College of Medicine, 103 Daehak-ro, Jongno-gu, Seoul, 110-799 South Korea; 3 Institute of Human Behavioral Medicine, Medical Research Center Seoul National University, Seoul, 110-799 South Korea; 4 Department of Neuropsychiatry, University of Ulsan College of Medicine, Ulsan University Hospital, Ulsan, 682-714 South Korea; 5 Department of Nuclear Medicine, Seoul Metropolitan Government–Seoul National University Boramae Medical Center, Seoul, 156-707 South Korea; 6 Department of Neuropsychiatry, Seoul National University Hospital, 101 Daehak-ro, Jongno-gu, Seoul, 110-744 South Korea; 7 Department of Psychiatry, Seoul National University College of Medicine, 101 Daehak-ro, Jongno-gu, Seoul, 110-744 South Korea

**Keywords:** Alzheimer’s disease, β-amyloid, Plasma Aβ, Blood-based biomarker, MPP, Pittsburgh-compound B positron emission tomography

## Abstract

**Background:**

Plasma β-amyloid (Aβ) is a potential candidate for an Alzheimer’s disease (AD) biomarker because blood is an easily accessible bio-fluid, which can be collected routinely, and Aβ is one of the major hallmarks of AD pathogenesis in the brain. However, the association between plasma Aβ levels and AD diagnosis is still unclear due to the instability and inaccurate measurements of plasma Aβ levels in the blood of patients with AD. If a consistent value of plasma Aβ from the blood can be obtained, this might help determine whether plasma Aβ is a potential biomarker for AD diagnosis.

**Methods:**

We predicted the brain amyloid deposit by measuring the plasma Aβ levels. This cross-sectional study included 353 participants (215 cognitively normal, 79 with mild cognitive impairment, and 59 with AD dementia) who underwent Pittsburgh-compound B positron emission tomography (PiB-PET) scans. We treated a mixture of protease inhibitors and phosphatase inhibitors (MPP) and detected plasma Aβ42 and Aβ40 (MPP-Aβ42 and MPP-Aβ40) in a stable manner using xMAP technology.

**Results:**

MPP-Aβ40 and MPP-Aβ42/40 (MPP-Aβs) were significantly different between subjects with positive amyloid deposition (PiB+) and those with negative amyloid deposition (PiB–) (*P* < 0.0001). Furthermore, MPP-Aβ40 (*P* < 0.0001, *r* = 0.23) and MPP-Aβ42/40 ratio (*P* < 0.0001, *r* = –0.23) showed significant correlation with global PiB deposition (standardized uptake value ratio). In addition, our integrated multivariable (MPP-Aβ42/40, gender, age, and apolipoprotein E genotypes) logistic regression model proposes a new standard for the prediction of cerebral amyloid deposition.

**Conclusions:**

MPP-Aβ might be one of the potential blood biomarkers for the prediction of PiB-PET positivity in the brain.

**Electronic supplementary material:**

The online version of this article (doi:10.1186/s13195-017-0248-8) contains supplementary material, which is available to authorized users.

## Background

Alzheimer’s disease (AD) is the most common cause of dementia usually observed in populations over the age of 65 years and is characterized by β-amyloid (Aβ) plaques and neurofibrillary tangles in the brain [1, 2]. The insoluble aggregates of Aβ peptides are associated with cognitive impairment, synapse loss, and neuronal cell death, and this may precede the onset of dementia [3, 4]. In addition, there is no currently available cure for AD, and current drugs only alleviate the symptoms of dementia but do not cure the underlying disease [5]. Abnormal aggregation of Aβ is the earliest pathological event in AD, and existing drugs only slow down AD progression. Hence, the diagnosis of AD at the early stages of the disease is the most promising method for effective follow-up measures, although it is very challenging. Furthermore, although AD has no known cure, early detection of the disease is promising because the devastating disease can be prevented through well-known methods for reducing risk factors such as doing some exercises, healthy diet, controlling blood pressure, having strong social support, taking folic acid supplements, and not smoking [6–11].

Efforts to identify the available and effective biomarkers for AD have been made in many countries. In the neuroimaging field, specific regional brain atrophy can be observed by magnetic resonance imaging (MRI), and Aβ deposition in the brain can be detected by positron emission tomography (PET) using specific radioactive ligands including Pittsburgh compound B (PiB) [12]. However, these are costly procedures for AD diagnosis [13]. Additionally, although molecular biomarkers such as tau and Aβ can be measured in the cerebrospinal fluid (CSF) [14], the collection of CSF is a highly invasive procedure [15].

On the other hand, the blood is an easily accessible bio-fluid which can be routinely collected and analyzed to detect or track the disease [15]. Hence, if we utilize blood proteins as a biomarker for AD, this might help recognize the disease earlier because of the simple method and high frequency of blood collection. Several studies reported the efflux/influx of Aβ across the blood–brain barrier and identified various Aβ transporters; hence, many researchers believe that plasma Aβ might reflect brain amyloid deposition [16]. However, the association between plasma Aβ levels and AD diagnosis is still unclear. Several cross-sectional studies showed that low plasma Aβ42 levels or Aβ42/40 ratios are associated with AD [17, 18]. Some prospective studies have also suggested that a decrease in plasma Aβ42/40 ratio is related to cognitive impairment [19, 20]. However, these results are in contrast with other reports, which indicate an increase in Aβ42 with cognitive decline [21], no association between plasma Aβ levels and AD [22], and a relation between low plasma Aβ40 and AD [23]. Thus, evidence for the effectiveness of measurement of plasma Aβ42, Aβ40, and the Aβ42/40 ratios in the diagnosis of AD is lacking. Such controversy on the relationship between plasma Aβ levels and AD may result from the inaccuracy in both plasma Aβ measurement and AD clinical diagnosis. In addition, plasma Aβ levels show unpredictable fluctuations in their values because of numerous factors. Human serum albumin (HSA) and transthyretin (TTR) interrupt the detection of Aβ [24–27] because both of them bind to Aβ, and their interaction status varies depending on blood dynamics [28, 29]. Furthermore, protease inhibitors and phosphatase inhibitors in the blood might play a role in decreasing Aβ degradation because the Aβ sequence contains many possible proteases and phosphorylation sites [30, 31]. After blood collection, both protease inhibitors and phosphatase inhibitors are randomly activated, which affects Aβ degradation and the interaction between Aβ and other proteins in the blood [31, 32], resulting in the fluctuation of the measurable Aβ concentration in the blood.

Moreover, about 12% of patients with clinically diagnosed AD dementia (ADD) do not exhibit AD pathology, and 23% of cognitively normal (CN) individuals show AD pathology [3, 33]. Hence, the blood samples from previous studies that did not have PiB-PET data for the participants were not homogeneous samples in the view of brain amyloid pathology, which might have yielded the conflicting results for plasma Aβ level.

In this study, we aimed to investigate the potential of plasma Aβ level as a diagnostic or screening marker for AD. To resolve the instability or inaccuracy in assessment, we applied two distinct approaches. First, all participants underwent PiB-PET to quantify the cerebral Aβ deposition. This approach allowed us to test the effectiveness of plasma Aβ as a biomarker using pathological AD diagnosis, instead of clinical AD diagnosis, as the gold standard. Second, we developed a novel mixture (MPP, a mixture of protease inhibitors and phosphatase inhibitors) and treated the plasma with MPP to detect plasma Aβ levels in a stable and accurate manner using xMAP technology. Under this new but simple method, we determined that MPP-Aβ might be a reliable blood biomarker for screening cerebral amyloid deposition.

## Methods

This study was part of the Korean Brain Aging Study for Early Diagnosis and Prediction of Alzheimer’s Disease (KBASE), an ongoing prospective cohort study aimed at searching for new biomarkers for AD and elucidating various life experiences contributing to AD-related brain changes. This work was approved by the Institutional Review Board (IRB) of the Seoul National University Hospital, South Korea, and the subjects or their legal representatives provided their written informed consent. Additional file 1 shows the experimental flow chart for this study.

### Participants

Overall 353 middle-aged or old-aged subjects with age ≥ 55 years, including 215 CN individuals, 79 individuals with mild cognitive impairment (MCI), and 59 individuals with ADD, participated in the study. All participants underwent comprehensive clinical and neuropsychological assessments, neuroimaging examinations including structural MRI and PiB-PET, and comprehensive laboratory blood tests. CN participants had a Clinical Dementia Rating (CDR) score of 0 [34] and were without diagnoses of MCI or dementia. Individuals with MCI met the following criteria: (a) memory complaint corroborated by self, an informant, or a clinician; (b) objective memory impairment for age, education, and gender; (c) largely intact functional activities; and (d) not demented. All individuals with MCI had a global CDR score of 0.5. In terms of criterion (b), all participants with MCI had a performance score at least 1.5 standard deviation (SD) below the respective age-specific, education-specific, and gender-specific mean for at least one of the four episodic memory tests included in the Consortium to Establish a Registry for Alzheimer’s Disease (CERAD) neuropsychological battery (namely, Word List Memory, Word List Recall, Word List Recognition, and Constructional Recall test) [35]. Patients with ADD met both the criteria for dementia (in accordance with the Diagnostic and Statistical Manual, fourth edition (DSM-IV)) [36] and the criteria for probable AD set in accordance with the National Institute of Aging and Alzheimer’s Association (NIA-AA) guidelines [37]. The exclusion criteria for all participants were: any present serious medical, psychiatric, and neurological disorders that could affect mental function; the presence of severe communication problems that would make a clinical examination or brain scan difficult; contraindications for MRI scan (e.g., pacemaker, claustrophobia); absence of a reliable informant; and illiteracy.

### Clinical and neuropsychological assessment

Participants were administered standardized clinical assessments based on the KBASE clinical assessment protocol, which incorporated the Korean version of the Consortium to Establish a Registry for Alzheimer’s Disease Assessment Packet (CERAD-K) [38], by trained psychiatrists. They were also administered the KBASE neuropsychological assessment protocol incorporating the CERAD neuropsychological battery [35], which included the Mini-Mental State Examination (MMSE), by trained neuropsychologists. In this study we used the MMSE *z* score, with consideration for age, gender, and education levels, because the basic demographic information of subjects such as gender, age, and education is known to have an effect on the change in MMSE scores [39].

### Blood sampling

Blood samples were obtained via venipuncture in the morning (around 9:00 am) after an overnight fast and collected several hours before the injection of PET tracer in K2 ethylenediaminetetraacetic acid (EDTA) tubes (BD Vacutainer Systems, Plymouth, UK). The samples were stabilized and centrifuged at 700 × *g* for 5 min at room temperature (RT) to obtain the plasma supernatants in 15-ml centrifuge tubes (SPL Life Sciences Co., Gyeonggi-do, Korea). To obtain the samples with high purity, the plasma supernatants were further centrifuged under the same conditions, and the collected pure plasma supernatants were aliquoted and stored immediately at –80 °C.

### PiB-PET

Participants underwent simultaneous three-dimensional PiB-PET imaging and T1-weighted MR using a Biograph mMR scanner (Siemens, Washington, DC, USA) with the manufacturer’s approved guidelines. A 30-min emission scan was acquired after 40 min of intravenous administration of 555 MBq of ^11^C-PiB (range, 450–610 MBq). The PiB-PET data collected in the list mode were processed for routine corrections such as uniformity, ultrashort echo time (UTE)-based attenuation, and decay corrections and were reconstructed into a 256 × 256 image matrix using iterative methods (six iterations with 21 subsets). T1-weighted scans (repetition time = 1670 ms; echo time = 1.89 ms; field of view = 250 mm; 256 × 256 matrix with 1.0 mm slice thickness) were acquired in the sagittal orientation. The following image preprocessing steps were performed using Statistical Parametric Mapping 8 (SPM8) implemented in MATLAB 2014a (MathWorks, Natick, MA, USA). Static PiB-PET images were coregistered to the individual T1 structural images and transformation parameters for spatial normalization of the individual T1 images to a standard Montreal Neurological Institute (MNI) template were calculated. Utilizing Individual Brain Atlases using Statistical Parametric Mapping Software (IBASPM) software, the inverse transformation parameters were used to transform the coordinates from the automatic anatomic labeling (AAL) 116 atlas [40] into an individual space for each subject (resampling voxel size = 1 × 0.98 × 0.98 mm^3^). The nongray matter portions of the atlas were individually masked using the cerebral gray matter segment image from each subject.

The mean regional ^11^C-PiB uptake values from the cerebral regions were extracted using the individual AAL116 atlas from the T1-coregistered PiB-PET images. The cerebellar gray matter was used as the reference region due to its relatively low Aβ deposition for the quantitative normalization of cerebral ^11^C-PiB uptake values. To measure the ^11^C-PiB uptake in the cerebellar gray matter regions, a probabilistic cerebellar atlas (Institute of Cognitive Neuroscience, UCL; Cognitive Neuroscience Laboratory, Royal Holloway) was transformed into an individual space in the same manner as already described. Of the 28 anatomical structural regions in the cerebellar atlas, all of the cerebellar lobular regions except for the vermis were included to extract the mean cerebellar uptake values. The AAL algorithm and a region-combining method [20] were applied to determine the regions of interest (ROIs) to characterize the ^11^C-PiB retention level in the frontal, lateral parietal, posterior cingulate-precuneus (PC-PRC), and lateral temporal regions, where prominent ^11^C-PiB retention has been reported [13].

The standardized uptake value ratio (SUVR) values for each ROI were calculated by dividing the mean value for all voxels within each ROI by the mean cerebellar uptake value in the same image. Each participant was classified as PiB-positive (PiB+) if the SUVR value was >1.4 in at least one of the four ROIs (i.e., frontal, lateral temporal, lateral parietal, and PC-PRC) or PiB-negative (PiB–) if the SUVR values of all four ROIs were ≤1.4 [17]. A global cortical ROI consisting of the four ROIs was also defined, and a global PiB deposition value (SUVR) was generated by dividing the mean value for all voxels of the global cortical ROI by the mean cerebellar uptake value of the same image.

### Reagents

The mixture of protease inhibitors and phosphatase inhibitors (MPP) was composed of protease inhibitor cocktail (PI), phenylmethanesulfonyl fluoride (PMSF, a serine protease inhibitor; Sigma-Aldrich, CA, USA), and phosphatase inhibitor cocktail I and II (PPI I and II; A. G. Scientific, Inc., CA, USA). They were mixed in the same proportion. Aβ peptide was purchased from Bachem Americas, Inc. (Torrance, CA, USA) and prepared as described previously [41]. HSA (Sigma-Aldrich) was used for mimicking the plasma sample condition because it is the most abundant protein in the human plasma [25, 42, 43].

### INNO-BIA plasma Aβ forms assay using xMAP technology

To simultaneously determine the concentrations of plasma Aβ42 and Aβ40, the INNO-BIA plasma Aβ forms kit (Innogenetics, Gent, Belgium) was used in accordance with the manufacturer’s guidelines. In brief, the samples were diluted 3-fold in the MPP-treated plasma diluent buffer (final concentration, 4% MPP solution in plasma diluent buffer) or MPP nontreated plasma diluent buffer and incubated for 30 min at RT. After washing the filter plate, the diluted bead mix was transferred to the wells of the plate. The plate was dried gently, washed, and 25 μl of conjugate 1 working solution A and 75 μl of standards, blanks, controls, and diluted plasma samples were added. The plate was incubated overnight at 4 °C, and the next day each well was washed with 100 μl of the detection solution added to the mixture. After 1 h, the plates were washed again, and the reading solution was added to each well. The levels of plasma Aβ were measured using xMAP technology (Bioplex 200 systems; Bio-Rad, Hercules, CA, USA).

### Proof-of-concept experiments

#### Gel electrophoresis

We prepared the synthetic Aβ42 (diluted in 1× phosphate buffered saline (PBS), 2 μM) to visualize the change in Aβ form against MPP treatment. Aβ42 solution was equally divided (20 μl) among eight 1.5 ml tubes. For repetitive measurement assay without plasma (pure synthetic Aβ42), 20 μl of 8% dimethyl sulfoxide (DMSO) in 1× PBS (–MPP) (*n* = 4; Fig. 2a, lanes 1–4) or 8% MPP solution in 1× PBS (+MPP) (*n* = 4; Fig. 2a, lanes 5–8) was added to the Aβ42 solution (final concentration: 1 μM of Aβ42). For repetitive measurement assay with the original plasma sample, 20 μl of 8% DMSO and 25% plasma in 1× PBS (–MPP) (*n* = 4; Fig. 2b, lanes 1–4) or 8% MPP solution and 25% plasma in 1× PBS (+MPP) (*n* = 4; Fig. 2b, lanes 5–8) was added to the Aβ42 solution (final concentration: 1 μM of Aβ42). The samples were incubated for 30 min at RT and then mixed with 4× sample buffer (without boiling) and loaded equally on 4–12% NuPAGE bis-tris gel (Thermo Fisher Scientific, Waltham, MA, USA) for gel electrophoresis. Next, the gel was transferred to a polyvinylidene difluoride (PVDF) membrane for 60 min at 70 V, and the membrane was blocked with 5% skim milk in 1× Tris buffered saline with Tween 20 (TBST) for 1 h. After blocking, the membrane was incubated with anti-6E10 Aβ antibody (primary antibodies overnight at 4 °C) and the following day with secondary antibodies for 1 h at RT. The protein bands on the PVDF membrane were visualized using a bio-imaging analyzer (LAS-3000; Fujifilm Corporation, Tokyo, Japan) with a chemiluminescence detection solution (Ab Frontier Co., Seoul, Korea). The images were imported from a Multi-Gauge program (Fujifilm Corporation), and the band intensities (ratio, %) were measured (monomeric Aβ, ~4 kDa; oligomeric Aβ, 8–14 kDa) [44].

#### xMAP technology

We repetitively measured the synthetic Aβ and plasma Aβ levels using xMAP technology. For synthetic Aβ levels, 4% MPP solution (or absent), 0.5% HSA (or absent), and Aβ42 (200 pg/ml) (aliquots from the same pool, in separate 1.5-ml tubes; –MPP or + MPP, each *n* = 6) in Bioplex sample diluent buffer were incubated for 30 min at RT, followed by Aβ42 measurement using xMAP technology. For plasma Aβ levels, samples (aliquots from the same pooled plasma, in separate 1.5-ml tubes, *n* = 5) were diluted 3-fold in MPP-treated plasma diluent buffer (final concentration, 4% MPP solution in plasma diluent buffer) or MPP nontreated plasma diluent buffer, incubated for 30 min at RT, and then measured using xMAP technology (–MPP or + MPP, each *n* = 5).

#### Time-dependent alterations in plasma Aβ42 and Aβ40 levels

Plasma Aβ42 and Aβ40 levels were quantified in a time-dependent manner using xMAP technology. We used individual human plasma samples (*n* = 4) and also diluted them 3-fold in MPP-treated plasma diluent buffer (final concentration, 4% MPP solution in plasma diluent buffer) or MPP nontreated plasma diluent buffer, and incubated at RT followed by measurement at each time point (0, 6, 12, 24 h) and quantification (–MPP or + MPP, each *n* = 4).

#### Effects of MPP on discrimination between subject groups

Plasma samples (20 CN–, 12 MCI+, 23 ADD+, total of 55 subjects; – and +, PiB-PET positivity) were diluted 3-fold in MPP-treated plasma diluent buffer (final concentration, 4% MPP solution in plasma diluent buffer) or MPP nontreated plasma diluent buffer and incubated for 30 min at RT followed by measurement using xMAP technology. We compared the intergroup differences in MPP-treated plasma Aβ (MPP-Aβ) with those of non-MPP-treated plasma Aβ (nMPP-Aβ).

### Statistics

Statistical analysis was performed using GraphPad Prism 5 (GraphPad Software, San Diego, CA, USA) and Medcalc (Medcalc Software, Ostend, Belgium). All data are presented as mean ± standard error of mean (SEM). An unpaired *t* test was used to assess the quantitative differences between two groups. For the tests of differences between three groups or more, multifactorial analyses of variance (ANOVA) followed by Tukey’s multiple comparisons tests were performed. We used a bivariate correlation analysis to test the association between variables. Furthermore, we conducted logistic regression analysis followed by receiver operating characteristic (ROC) curve analysis on these values using Medcalc software. Cutoff criteria (optimal cutoff point) were determined by the Youden index [45] with an appropriate balance between sensitivity and specificity. Pearson’s chi-square test (χ^2^) was carried out to evaluate the intergroup differences of two-category variables (gender, apolipoprotein E ε4 carrier status) using Medcalc software. To compare the variance between –MPP and + MPP in the repetitive measurement assay, an *F* test was performed (GraphPad Prism 5). Squared deviation ((value – mean value)^2^) indicates how far each point is from the mean value. Because the *y* axis of the graphs indicates the squared deviation in Fig. 2a–d, the highest points of each bar graph equates to ‘Variance’:

$$ \mathrm{Variance}=\sum \left\{{\left( X{\textstyle \hbox{--} } m\right)}^2/ N\right\}, $$

where *X* is an individual data point, *m* is the mean of data points, and *N* is the total number of data points. Statistically significant results were shown as the appropriate *P* value.

## Results

### Demographic data of subjects

**Fig. 1:**
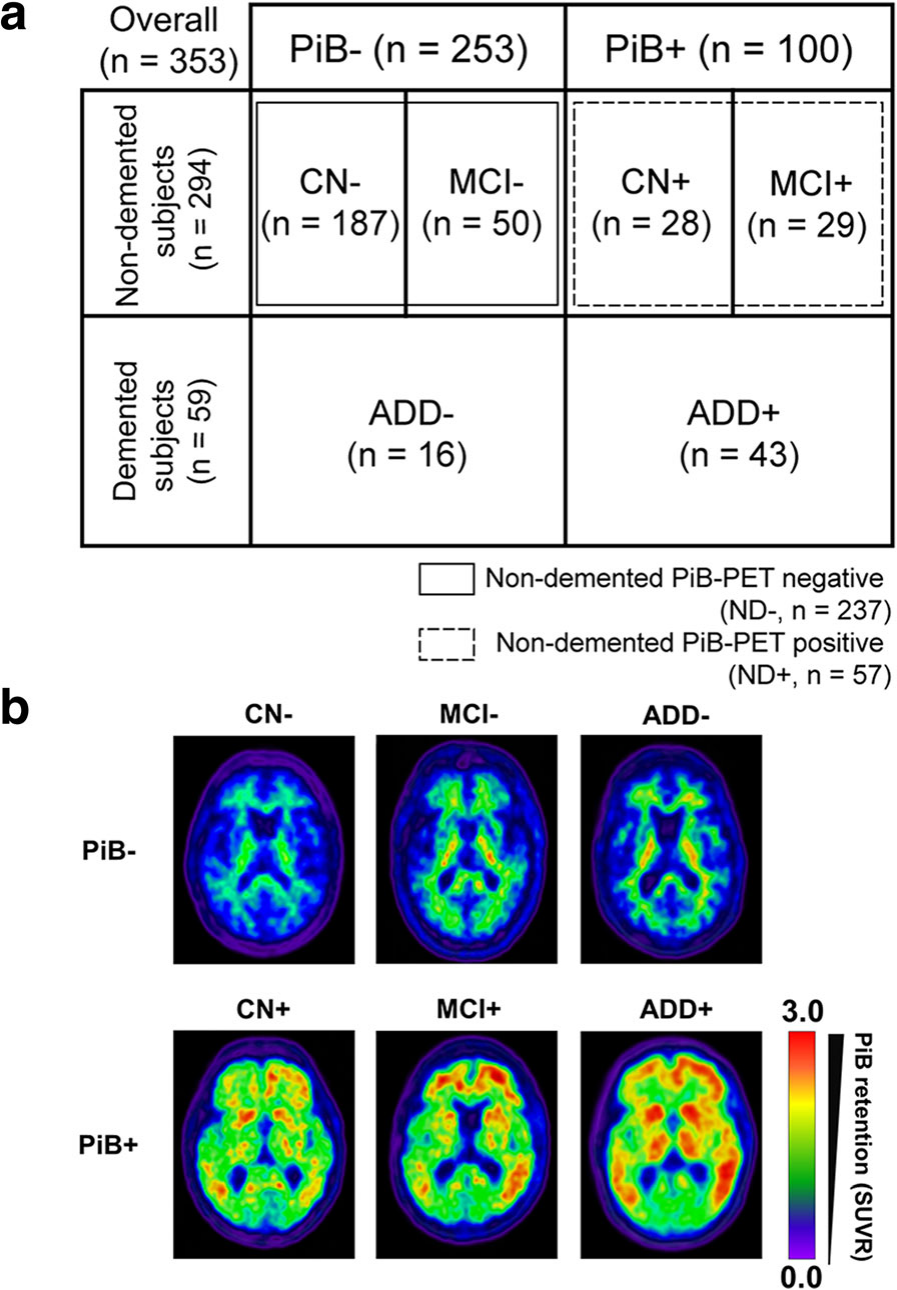
Categorized subject groups. **a** Classification of subjects (*n* = 353) for the study. **b** Representative PiB-PET images of the study cohort (*n* = 353). Participants were classified as PiB-positive (*PiB+*) if the PiB retention (SUVR) value was >1.4 in at least one of the four ROIs (i.e., frontal, lateral temporal, lateral parietal, and PC-PRC) or PiB-negative (*PiB–*) if the SUVR values of all four ROIs were ≤1.4. *– or +* PiB-PET positivity, *CN* cognitively normal, *MCI* mild cognitive impairment, *ADD* Alzheimer’s disease dementia, *ND* nondemented, *PiB-PET* Pittsburgh-compound B positron emission tomography, *SUVR* standard uptake value ratio (Color figure online)

A total of 353 subjects were included in this study. Subjects were classified according to their pathological (brain Aβ deposition) and/or clinical diagnosis (Fig. 1, Table 1, and Additional file 2). Two hundred and fifty-three subjects with negative amyloid deposition (PiB–) and 100 subjects with positive amyloid deposition (PiB+) were included; out of which 187 were PiB– CN (CN–), 50 were PiB– MCI (MCI–), 16 were PiB– ADD (ADD–), 28 were PiB+ CN (CN+), 29 were PiB+ MCI (MCI+), and 43 were PiB+ ADD (ADD+). Figure 1b shows the representative PiB-PET images of the study cohort (*n* = 353). The colors of the rainbow spectrum show the degree of Aβ deposition (PiB retention, SUVR; red → purple, high → low, respectively).

**Table 1:** Demographic data of the complete study cohort for the main experiments (PiB– vs PiB+)

	Group (total, *n* = 353)		
Basic characteristic	PiB– (*n* = 253)	PiB+ (*n* = 100)	*P* value^a^
Global PiB deposition, mean ± SEM	1.11 ± 0.004	1.91 ± 0.038	<0.001
Clinical diagnosis, N/M/D	187/50/16	28/29/43	<0.001^†^
Gender, male/female	95/158	38/62	>0.05^†^
Age (years), mean ± SEM	69.94 ± 0.5	73.00 ± 0.7	<0.001
CDR, mean ± SEM	0.15 ± 0.02	0.5 ± 0.04	<0.001
MMSE *z* score,^b^ mean ± SEM	–0.13 ± 0.07	–1.91 ± 0.20	<0.001
Education, mean ± SEM	10.65 ± 0.3	10.82 ± 0.5	>0.05
ApoE4-positive, *n*/*N* (%)	39/253 (15%)	57/100 (57%)	<0.001^†^

Details of clinical diagnosis and pathological states (CDR score, MMSE *z* score, and global PiB deposition (SUVR)) of each subject are shown in Additional file 3

*PiB* Pittsburgh-compound B, *SUVR* standardized uptake value ratio, *– or +* PiB-PET positivity, *MMSE* Mini-Mental State Examination, *CDR* Clinical Dementia Rating, *ApoE* apolipoprotein E, *SEM* standard error of mean, *n* number of subjects, *N/M/D* cognitively normal/mild impairment/dementia

^a^ *P* value, significance by unpaired *t*-test except for clinical diagnosis, gender, and ApoE

^b^A revised value of the MMSE score with consideration for age, gender, and education level

^†^Pearson’s chi-square test

### Proof-of-concept experiments for the effects of MPP on Aβ quantification

**Fig. 2:**
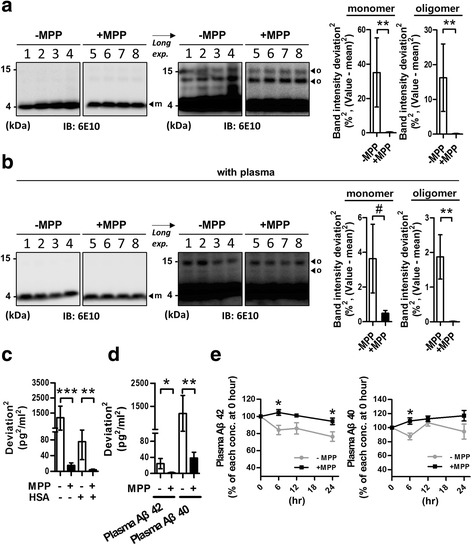
POC for the effects of MPP on Aβ quantification. **a** Gel electrophoresis for synthetic Aβ42 form assay. Twenty microliters of 8% DMSO in 1× PBS (–MPP) or 20 μl of 8% MPP solution in 1× PBS (+MPP) was added to 20 μl of 2 μM Aβ42 (final concentration, 1 μM; aliquots from the same pool, in separate 1.5-ml tubes, *n* = 4), and the samples were electrophoresed. Band intensity squared deviation ((value – mean value)^2^) indicates how far each point is from the mean value (**P < 0.01, *F* test to compare variances; each *n* = 4). **b** Gel electrophoresis for synthetic Aβ42 form assay with the original plasma sample. Twenty microliters of 8% DMSO and 25% plasma in 1× PBS (–MPP) or 20 μl of 8% MPP and 25% plasma in 1× PBS (+MPP) was added to 20 μl of 2 μM Aβ42 (final concentration, 1 μM; aliquots from the same pool, in separate 1.5-ml tubes, *n* = 4), and the samples were electrophoresed. Band intensity squared deviation indicates how far each point is from the mean value (^#^ *P* < 0.1, a trend toward significance; **P < 0.01, *F* test to compare variances; each *n* = 4). **c** Repetitive measurement of synthetic Aβ42 (aliquots from the same pool, in separate 1.5-ml tubes, *n* = 6) using xMAP technology, with or without MPP and/or HSA. HSA was used for mimicking blood plasma condition. Squared deviation indicates how far each point is from the mean value (**P < 0.01 and ***P < 0.001, *F* test to compare variances). **d** Repetitive measurement (aliquots from the same pooled plasma, in separate 1.5-ml tubes, *n* = 5) of plasma Aβ42 and Aβ40 using xMAP technology, with or without MPP. Squared deviation indicates how far each point is from the mean value (*P < 0.05 and **P < 0.01, *F* test to compare variances). **e** Quantification of plasma Aβ42 and Aβ40 in a time-dependent manner using xMAP technology, with or without 4% MPP solution in Bioplex sample diluent buffer (*n* = 4, independent individual plasma samples; *P < 0.05, unpaired *t* test for –MPP vs + MPP at each time point). *m* monomeric Aβ; *o* oligomeric Aβ, *Aβ* β-amyloid, *MPP* mixture of protease inhibitors and phosphatase inhibitors, *HSA* human serum albumin

To verify the effect of MPP on Aβ quantification, we used the synthetic Aβ42 and plasma samples for the proof-of-concept experiments (POC) (Fig. 2). When the same concentration of synthetic Aβ42 (final concentration: 1 μM of Aβ42) in the presence or absence of MPP was electrophoresed using western blotting, the Aβ42 samples without MPP treatment showed variable band intensities among all four lanes of the blot, whereas MPP-treated Aβ42 samples presented consistent band intensities in the blot (Fig. 2a; *P* < 0.01, *F* test; for monomeric Aβ, variance decreased by 34.75%^2^; for oligomeric Aβ, variance decreased by 16.11%^2^). Similarly, this phenomenon occurred when the original plasma sample was added (Fig. 2b; *P* < 0.1 and *P* < 0.01, *F* test; for monomeric Aβ, variance decreased by 3.16%^2^; for oligomeric Aβ, variance decreased by 1.86%^2^). Next, we treated synthetic Aβ42 with MPP and/or HSA and measured the Aβ42 level using xMAP technology. Interestingly, the repetitively measured Aβ42 concentrations showed dramatically reduced variance among their values for the MPP-treated sample set (Fig. 2c; *P* < 0.001, without HSA, variance decreased by 1190.6 pg^2^/ml^2^; *P* < 0.01 with HSA, variance decreased by 70.99 pg^2^/ml^2^; *F* test). Furthermore, the repetitive measurement of plasma Aβ42 and Aβ40 showed significantly reduced variation (Fig. 2d; plasma Aβ42, *P* < 0.05, variance decreased by 22.70 pg^2^/ml^2^; plasma Aβ40, *P* < 0.01, variance decreased by 1151.34 pg^2^/ml^2^; *F* test). In addition, we identified whether MPP could inhibit the degradation of plasma Aβ over time (from 0 to 24 h) because a previous study reported that plasma sample storage at RT leads to a significant loss of measurable Aβ peptide level [46]. For the human plasma samples, MPP-treated plasma Aβ42 (MPP-Aβ42) and MPP-Aβ40 remained stable for 24 h whereas non-MPP-treated plasma Aβ42 (nMPP-Aβ42) levels decreased rapidly, and nMPP-Aβ40 levels fluctuated (Fig. 2e, *P* < 0.05, unpaired *t* test at each time point). This result indicates that MPP has an effect on stabilizing plasma Aβ for 24 h.

### POC for the effects of MPP on the distinction among the subject groups

**Fig. 3:**
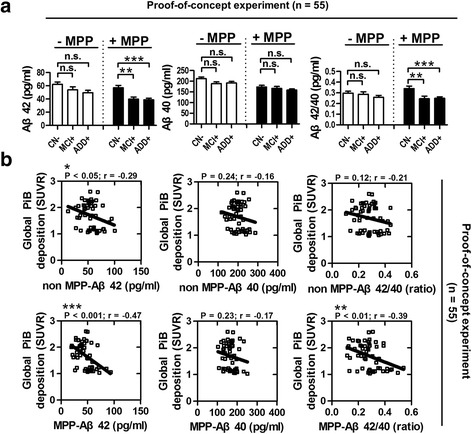
POC for the effect of MPP on distinction between subjects. **a** Intergroup differences in the plasma Aβ concentration with or without 4% MPP solution using xMAP technology. MCI+ and ADD+ subjects show significantly decreased levels of MPP-Aβ42 and MPP-Aβ42/40 ratio compared with CN– subjects (**P < 0.01 and ***P < 0.001 respectively; ANOVA followed by Tukey’s multiple comparison test; CN–, *n* = 20; MCI+, *n* = 12; ADD+, *n* = 23; total subjects, *n* = 55). **b** Correlation of global PiB deposition (SUVR) and MPP-Aβs. MPP-Aβ42 level (***P < 0.001, *r* = –0.47; Pearson’s correlation) and MPP-Aβ42/40 ratio (**P < 0.01, *r* = –0.39; Pearson’s correlation) are correlated with global PiB deposition (SUVR). *Aβ* β-amyloid, *MPP* mixture of protease inhibitors and phosphatase inhibitors, *MCI* mild cognitive impairment, *ADD* Alzheimer’s disease dementia, *CN* cognitively normal, *SUVR* standard uptake value ratio, *PiB* Pittsburgh compound B, *– or +* PiB negativity or positivity

To examine the correlation between the plasma Aβ concentration and brain Aβ deposition, plasma samples from 55 subjects (20 CN–, 12 MCI+, 23 ADD+; see Additional file 4 for more detail) who received PiB-PET imaging were used for further analysis (Fig. 3a, b). nMPP-Aβ42 and nMPP-Aβ42/40 levels showed no significant intergroup differences among all groups. In contrast, MPP-Aβ42 and MPP-Aβ42/40 were significantly lower in MCI+ subjects (MPP-Aβ42, 39.76 ± 3.26 pg/ml; MPP-Aβ42/40, 0.24 ± 0.02) and ADD+ subjects (MPP-Aβ42, 38.65 ± 2.45 pg/ml; MPP-Aβ42/40, 0.25 ± 0.02) compared with CN– subjects (MPP-Aβ42, 56.98 ± 3.57 pg/ml; MPP-Aβ42/40, 0.34 ± 0.02) (Fig. 3a; *P* < 0.01 and *P* < 0.001, ANOVA followed by Tukey’s multiple comparison test). Interestingly, when global PiB deposition values (SUVR) were plotted with plasma Aβ concentration, MPP-Aβ42 and MPP-Aβ42/40 (Fig. 3b; MPP-Aβ42, *P* < 0.001, *r* = –0.47; MPP-Aβ42/40, *P* < 0.01, *r* = –0.39; Pearson’s correlation) were more correlated with brain amyloid deposit than nMPP-Aβ42 and nMPP-Aβ42/40 (Fig. 3b; nMPP-Aβ42, *P* < 0.05, *r* = –0.29; nMPP-Aβ42/40, *P* = 0.12, *r* = –0.21; Pearson’s correlation). Therefore, we suggest that the treatment of the plasma with MPP provides an improved detection method for stable and reliable plasma Aβ level, and plasma Aβ concentration might reflect the brain amyloid deposit.

### MPP-Aβs reflect the pathological load of Aβ in the brain

**Fig. 4:**
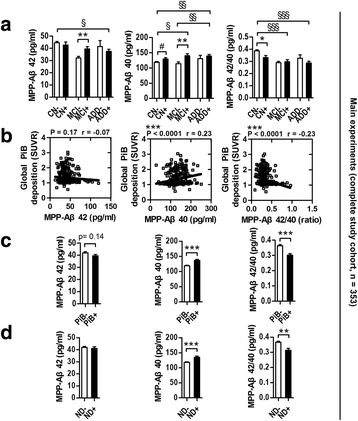
MPP-Aβs reflect the pathological load of Aβ in the brain. **a** Intergroup differences of MPP-Aβ levels using xMAP technology (*P < 0.05 and **P < 0.01, unpaired *t* test; ^§^ *P* < 0.05, ^§§^ *P* < 0.01, and ^§§§^ *P* < 0.001, ANOVA followed by Tukey’s multiple comparison test; ^#^ *P* < 0.10, unpaired *t* test, trend toward significance; CN–, *n* = 187, CN+, *n* = 28, MCI–, *n* = 50, MCI+, *n* = 29, ADD–, *n* = 16, ADD+, *n* = 43; total subjects *n* = 353). **b** Correlation of global PiB deposition (SUVR) and MPP-Aβs (*n* = 353, ***P < 0.0001; Pearson’s correlation). **c**, **d** MPP-Aβ42, MPP-Aβ40, and MPP-Aβ42/40 levels in ND– (*n* = 237; CN– and MCI–), ND+ (*n* = 57; CN+ and MCI+), PiB– (*n* = 253; CN–, MCI–, and ADD–), and PiB+ (*n* = 100; CN+, MCI+, and ADD+) (**P < 0.01, and ***P < 0.001, unpaired *t* test). *Aβ* β-amyloid, *MPP* mixture of protease inhibitors and phosphatase inhibitors, *MCI* mild cognitive impairment, *ADD* Alzheimer’s disease dementia, *CN* cognitively normal, *SUVR* standard uptake value ratio, *PiB* Pittsburgh compound B, *– or +* PiB negativity or positivity, *ND* nondemented

In the POC, we demonstrated that MPP works efficiently to improve stability in the quantification of plasma Aβ. To examine further the association of MPP-Aβ levels with the progression of AD, we conducted the main experiments by increasing the number of subjects (overall 353 subjects: 187 CN–, 50 MCI–, 16 ADD–, 28 CN+, 29 MCI+, and 43 ADD+; Fig. 4). Likewise with POC results, CN– subjects had higher MPP-Aβ42 (44.57 ± 1.05 pg/ml) concentration than ADD+ subjects (37.50 ± 1.72 pg/ml) (*P* < 0.05, Fig. 4a, left graph; ANOVA followed by Tukey’s multiple comparison test) and had higher levels of MPP-Aβ42/40 (0.38 ± 0.01) than both MCI+ (0.29 ± 0.02) and ADD+ (0.28 ± 0.01) subjects (*P* < 0.001, Fig. 4a, right graph; ANOVA followed by Tukey’s multiple comparison test). For further study, we performed a bivariate correlation analysis to test the relationship between MPP-Aβ level and global PiB deposition (SUVR) indicating the degree of cortical Aβ plaque deposition (Fig. 4b and Table 2) and compared PiB– and PiB+ subjects (Fig. 4c). First, we performed the Pearson’s correlation analysis for various groups. The MPP-Aβ40 level and the MPP-Aβ42/40 ratio showed significant correlation with global PiB deposition (SUVR) (MPP-Aβ40, *P* < 0.0001, *r* = 0.23; MPP-Aβ42/40 ratio, *P* < 0.0001, *r* = –0.23; Fig. 4b and Table 2) in the complete study cohort (all, *n* = 353). Furthermore, there were several significant correlations in many subgroups as well as in the overall study group. Both MPP-Aβ42 (*P* < 0.01, *r* = 0.3479) and MPP-Aβ40 (*P* < 0.01, *r* = 0.2950) levels were dramatically correlated with global PiB deposition (SUVR) in MCI subjects (*n* = 79). The MPP-Aβ40 level was significantly associated with global PiB deposition (SUVR) in nondemented subjects (ND = CN plus MCI, *n* = 294) (*P* < 0.001, *r* = 0.2177), in cognitively impaired subjects (CI = MCI plus ADD, *n* =138) (*P* < 0.01, *r* = 0.2272), and in CN subjects (*n* = 215) (*P* < 0.05, *r* = 0.1479) (Table 2). The MPP-Aβ42/40 ratio was correlated with global PiB deposition (SUVR) in ND subjects (*P* < 0.01, *r* = –0.1654). Furthermore, the MPP-Aβ42/40 ratio (*P* = 0.09, *r* = –0.1145) had a trend toward significant correlation with global PiB deposition (SUVR) in CN subjects (Table 2). This suggests that MPP-Aβ levels reflect the state of Aβ plaque deposition in the brain. Table 2 presents the detailed information of the correlation between global PiB deposition (SUVR) and MPP-Aβ levels. Next, we found that PiB– subjects (CN– plus MCI– plus ADD–, *n* = 253) had significantly different levels of MPP-Aβ40 (118.70 ± 2.09 pg/ml) and MPP-Aβ42/40 ratio (0.36 ± 0.01) in comparison with PiB+ subjects (CN+ plus MCI+ plus ADD+, *n* = 100; MPP-Aβ40, 136.60 ± 3.37 pg/ml; MPP-Aβ42/40, 0.30 ± 0.01) (Fig. 4c, *P* < 0.0001, unpaired *t* test). In addition, the MPP-Aβ40 level and MPP-Aβ42/40 ratio were dramatically different in PiB+ ND subjects (ND+, *n* = 57) (MPP-Aβ40, 134.80 ± 4.10 pg/ml; MPP-Aβ42/40, 0.31 ± 0.01) compared with PiB– ND subjects (ND–, *n* = 237) (MPP-Aβ40, 117.80 ± 2.10 pg/ml; MPP-Aβ42/40, 0.36 ± 0.01) (Fig. 4d, *P* < 0.01 and *P* < 0.0001, unpaired *t* test). Furthermore, MCI+ subjects showed significantly higher MPP-Aβ42 (39.46 ± 1.88 pg/ml) and MPP-Aβ40 (140.00 ± 6.86 pg/ml) levels than MCI– subjects (MPP-Aβ42, 32.03 ± 1.37 pg/ml; MPP-Aβ40, 114.30 ± 6.86 pg/ml) (*P* < 0.01, Fig. 4a, left and middle graphs, unpaired *t* test). Moreover, CN+ subjects showed significantly lower values of MPP-Aβ42/40 ratio (0.33 ± 0.02) than CN– subjects (0.39 ± 0.01) (*P* < 0.05, Fig. 4a, right graph, unpaired *t* test) and higher values of MPP-Aβ40 (129.40 ± 4.26 pg/ml) than CN– subjects (118.80 ± 2.24 pg/ml) (*P* = 0.08, a trend toward significance; Fig. 4a, middle graph, unpaired *t* test).

**Table 2:** Detailed information of the correlation between brain amyloid deposition (global PiB deposition (SUVR)) and MPP-Aβs

	CN	MCI	ADD	ND	CI	PiB–	PiB+	All
	(*n* = 215)	(*n* = 79)	(*n* = 59)	(*n* = 294)	(*n* = 138)	(*n* = 253)	(*n* = 100)	(*n* = 353)
MPP-Aβ42	*P* = 0.7982	**P < 0.01	*P* = 0.3132	*P* = 0.9827	*P* = 0.1125	*P* = 0.9750	*P* = 0.7965	*P* = 0.1776
	*r* = 0.0175	*r* = 0.3479	*r* = –0.1336	*r* = –0.0012	*r* = 0.1357	*r* = 0.0020	*r* = –0.0261	*r* = –0.0719
MPP-Aβ40	*P = 0.0301	**P < 0.01	*P* = 0.6639	***P < 0.001	**P < 0.01	*P* = 0.8155	*P* = 0.2436	****P < 0.0001
	*r* = 0.1479	*r* = 0.2950	*r* = 0.0578	*r* = 0.2177	*r* = 0.2272	*r* = –0.0147	*r* = 0.1177	*r* = 0.2309
MPP-Aβ42/40	^#^ *P* = 0.0940	*P* = 0.6395	*P* = 0.2868	**P < 0.01	*P* = 0.6488	*P* = 0.7416	*P* = 0.4437	****P < 0.0001
	*r* = –0.1145	*r* = 0.0535	*r* = –0.1410	*r* = –0.1654	*r* = –0.0391	*r* = –0.0208	*r* = –0.0775	*r* = –0.2280

*SUVR* standard uptake value ratio, *MPP-Aβ* MPP-treated plasma β-amyloid, *CN* cognitively normal, *MCI* mild cognitive impairment, *ADD* Alzheimer’s disease dementia, *ND* nondemented group (CN plus MCI), *CI* cognitively impaired group (MCI plus ADD), *PiB– or PiB+* subjects with negative or positive amyloid deposition, *All* complete study cohort

*P < 0.05, **P < 0.01, ***P < 0.001, and ****P < 0.0001 and Pearson *r* values by Pearson’s correlation analysis (two-tailed)

^#^ *P* < 0.10, trend toward significance

### Prediction of cerebral amyloid deposition based on MPP-Aβ42/40 ratio

**Fig. 5:**
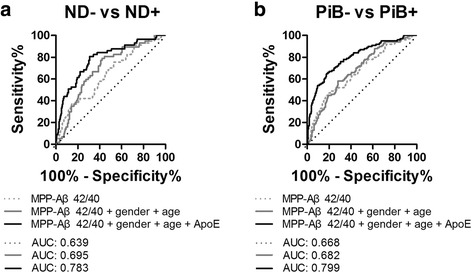
Logistic regression model and ROC curve analysis for PiB-PET prescreening ROC curve model using a combination of variables (MPP-Aβs, gender, age, and ApoE types) following the logistic regression analysis comparing (**a**) ND– and ND+ or (**b**) PiB– and PiB+. See details in Table 3. *PiB* Pittsburgh compound B, *– or +* PiB negativity or positivity, *ND* nondemented, *ApoE* apolipoprotein E, *MPP-Aβ* MPP-treated plasma β-amyloid, *AUC* area under curve

To apply these analyzed results (Fig. 4) to practical use, we performed a subsequent logistic regression and ROC curve analysis using independent variables (Fig. 5). Multiple variables (MPP-Aβs and ApoE genotype) and control variables (gender and age) were mixed by logistic regression analysis, and the predicted probabilities were used to assess the discrimination power (ROC curve analysis) for the prediction of PiB-PET positivity. The classification variable was ND– vs ND+ (Fig. 5a) or PiB– vs PiB+ (Fig. 5b). The combination of MPP-Aβ42/40 and control variables (gender and age) increased the area under curve (AUC) values (ND– vs ND+, 0.695; PiB– vs PiB+, 0.682) compared with MPP-Aβ42/40 alone (ND– vs ND+, 0.639; PiB– vs PiB+, 0.668) (Fig. 5 and Table 3). Furthermore, AUC values were further enhanced on adding the ApoE variable (ND– vs ND+, 0.783; PiB– vs PiB+, 0.799). Table 3 presents more detail about the results of logistic regression followed by ROC curve analysis.

**Table 3:** Detailed information of ROC curve analysis

Panel	AUC	Sensitivity (%)	Specificity (%)	*P* value	Cutoff criterion	95% CI of AUC
ND– vs ND+						
MPP-Aβ42/40	0.639	71.8	51.5	<0.0001	>0.1871	0.559–0.719
MPP-Aβ42/40 + age + gender	0.695	79.0	57.8	<0.0001	>0.1864	0.622–0.767
MPP-Aβ42/40 + age + gender + ApoE	0.783	80.7	69.2	<0.0001	>0.1730	0.715–0.852
PiB– vs PiB+						
MPP-Aβ42/40	0.668	71.0	52.2	<0.0001	>0.2731	0.605–0.731
MPP-Aβ42/40 + age + gender	0.682	71.0	54.2	<0.0001	>0.2759	0.622–0.741
MPP-Aβ42/40 + age + gender + ApoE	0.799	78.0	66.8	<0.0001	>0.2230	0.746–0.852

*ApoE* Apolipoprotein E, *AUC* area under curve, *MPP* mixture of protease inhibitors and phosphatase inhibitors, *CI* confidence interval, *ROC* receiver operating characteristic, *– or +* PiB-PET positivity, *ND–* PiB-PET-negative cognitively normal (*CN*) and PiB-PET-negative subjects with mild cognitive impairment (*MCI*), *ND+* PiB-PET-positive CN and PiB-PET-positive MCI, *PiB–* grouped CN–, MCI–, and ADD–, *PiB+* grouped CN+, MCI+, and ADD+

## Discussion

Plasma Aβ levels are believed unstable, and many plasma proteins such as HSA, TTR, and others are believed to interrupt the detection of Aβ [24, 26]. Furthermore, the plasma contains diverse proteases released from neutrophils and phagocytes [40, 47, 48]. Although the plasma contains several protease inhibitors such as α_2_-macroglobulin, α_1_-protease inhibitor, and plasminogen activator inhibitor-1 (PAI-1) [49–52], these are not sufficient for the preservation of the integrity of plasma proteins during the storage of samples at 4 °C or RT [53]. Moreover, Kumar et al. [31] reported that phosphorylation of Aβ at Ser-8 inhibited the degradation of monomeric Aβ. Accurate analysis with plasma requires a high quality of starting samples; however, there have been no previous attempts to stabilize the plasma Aβ by chemical treatment. We hypothesized that protease inhibitors and phosphatase inhibitors might have a role in reducing Aβ degradation because the Aβ sequence contains many possible proteases and phosphorylation sites [30, 31]. We found that the variation in Aβ concentration through repetitive measurement (using western blot and Luminex system) was significantly reduced by MPP treatment in a variety of settings including Aβ with pure sample diluent buffer (1× PBS or Bioplex buffer) (Fig. 2a, c), with plasma mimic buffer using HSA (Fig. 2c) [43], and with diluent buffer containing the original plasma sample (Fig. 2b, d). The purpose of repetitively measuring experiments (Fig. 2a–d) was to investigate whether the same samples distributed in different tubes show the same concentration value. Because of the instability of Aβ peptides, the detected values might have changed (during the incubation for 30 min at RT) depending on the independent tubes, even though they were same samples. As expected, without MPP treatment, the detected values fluctuated; however, when we treated samples with MPP, the detected values were quite similar to each other. This indicates that MPP allows us to control the unavoidable experimental errors (especially unexpected time delay) that occur during the experiment procedure. Furthermore MPP-treated plasma Aβ42 and Aβ40 (MPP-Aβ42 and MPP-Aβ40) maintained their concentration efficiently for 24 h whereas non-MPP-treated plasma Aβ42 (nMPP-Aβ42) levels decreased rapidly, and nMPP-Aβ40 levels fluctuated with incubation times (Fig. 2e). These results indicate that MPP might have a stabilizing effect on the quantification of plasma Aβs. Hence, we next differentiated PiB– (CN–, MCI–, and ADD–) vs PiB+ (CN+, MCI+, and ADD+) subjects using MPP-Aβs. Interestingly, although not all comparisons were statistically significant, most cases comparing PiB– vs PiB+ had the same patterns for MPP-Aβ40 level and MPP-Aβ42/40 ratio (MPP-Aβ40, CN– < CN+, MCI– < MCI+, and ADD– < ADD+; MPP-Aβ42/40, CN– > CN+, and ADD– > ADD+; Fig. 4a). Hence, we compared the subjects again as ND– vs ND+ and PiB– vs PiB+. The MPP-Aβ40 level was dramatically increased, and the MPP-Aβ42/40 ratio was significantly reduced in ND+ and PiB+ in comparison with ND– and PiB– subjects respectively (Fig. 4c, d). This indicates that MPP-Aβ might be a potential biomarker to predict Aβ deposition in the brain. Although the age of subjects who were PiB+ was relatively higher than that of PiB– subjects (Table 1), the significant difference of MPP-Aβs between PiB+ and PiB– subjects (MPP-Aβ40 increased and MPP-Aβ42/40 decreased in PiB+, compared with PiB–; Fig. 4c) was not due to the effects of aging because comparing young-middle-aged controls (YC, cognitively normal subjects with age 20–55 years, *n* = 61) vs CN– subjects showed completely opposite trends to PiB– vs PiB+ subjects, regarding MPP-Aβs (see Additional files 5 and 6 for more detail).

Even in cases where the MPP-Aβ42/40 ratio was dramatically different between PiB– and PiB+ subjects, it was insufficient to be used as a variable for ROC curve analysis to discriminate ND– vs ND+ or PiB– vs PiB+ subjects (ND– vs ND+, AUC 0.639 with 71.8% sensitivity and 51.5% specificity; PiB– vs PiB+, AUC 0.668 with 71.0% sensitivity and 52.2% specificity; Fig. 5 and Table 3). Using the combination of additional risk factors (gender, age, and ApoE types) reported previously [54, 55], we conducted the logistic regression analysis followed by ROC curve analysis to establish the screening model for cerebral amyloid deposition. We showed that the combination of MPP-Aβ42/40 ratio, gender, and age had a stronger discrimination power (ND– vs ND+, AUC 0.695 with 79.0% sensitivity and 57.8% specificity; PiB– vs PiB+, AUC 0.682 with 71.0% sensitivity and 54.2% specificity; Fig. 5 and Table 3) than the MPP-Aβ42/40 level alone. Furthermore, we observed that AUC was increased further by ApoE variable (ND– vs ND+, AUC 0.783 with 80.7% sensitivity and 69.2% specificity; PiB– vs PiB+, AUC 0.799 with 78.0% sensitivity and 66.8% specificity; Fig. 5 and Table 3).

These results are in line with several previous reports that showed an interrelationship between plasma Aβ42/40 ratio and dementia [56, 57]. However, this study is the first to stabilize the quantification of plasma Aβ by the treatment of chemicals and predict the PiB-PET positivity by classifying the subjects into two large groups, ND– vs ND+ subjects and PiB– vs PiB+ subjects, regardless of the stage of cognitive impairment. We showed a distinct relationship between MPP-Aβs and pathological AD-related Aβ burden in the brain. In conclusion, these results suggest that our model using MPP-Aβs and other factors could be utilized as a prescreening tool to predict cerebral Aβ deposition.

## Conclusions

The treatment of the plasma with a mixture of protease inhibitors and phosphatase inhibitors (MPP) provides an improved detection method for reliable plasma Aβ level, and MPP-Aβs showed a correlation with Aβ burden in the brain.

## Additional files

Additional file 1: is a figure showing the experimental flow chart. (DOCX 37 kb)

Additional file 2 is a table presenting demographic data of the complete study cohort. (DOCX 58 kb)

Additional file 3: is a figure showing clinical and pathological states of the study cohort: (**a**) SUVR of subjects (*P < 0.05 and ***P < 0.001, ANOVA followed by Tukey’s multiple comparison test), (**b**) MMSE *z* score (*P < 0.05, ANOVA followed by Tukey’s multiple comparison test), and (**c**) CDR score (***P < 0.001, ANOVA followed by Tukey’s multiple comparison test). (DOCX 67 kb)

Additional file 4: is a table presenting demographic data of subjects for Proof-of-concept (POC) experiments in the study. (XLSX 11 kb)

Additional file 5: is a figure showing MPP-Aβs and aging: (**a**) CN– show significantly higher MPP-Aβ42 and MPP-Aβ42/40 ratio than YC (***P < 0.001, unpaired *t* test) and (**b**) MPP-Aβ42 and MPP-Aβ42/40 ratio show significant association with aging (***P < 0.0001, *r* = 0.40 for MPP-Aβ42; ***P < 0.0001, *r* = 0.33 for MPP-Aβ42/40 ratio; Pearson’s correlation). (XLSX 11 kb)

Additional file 6: is a table presenting demographic data of young-middle-aged controls. (XLSX 10 kb)

## Abbreviations

ADD: Alzheimer’s disease dementia
AUC: Area under curve
Aβ: β-Amyloid
CN: Cognitively normal
MCI: mild cognitive impairment
MMSE: Mini-Mental State Examination
MPP: Mixture of protease inhibitors and phosphatase inhibitors
MPP-Aβ: MPP-treated plasma Aβ
ND–: Nondemented PiB-PET-negative
ND+: Nondemented PiB-PET-positive
PiB–: Negative amyloid deposition
PiB+: Positive amyloid deposition
PiB-PET: Pittsburgh-compound B positron emission tomography
POC: Proof-of-concept experiments
SUVR: Standardized uptake value ratio
