# Supplementary material for: Chemically treated plasma Aβ is a potential blood-based biomarker for screening cerebral amyloid deposition
Source: Alzheimers Res Ther. 2017 Mar 22;9:20. doi: 10.1186/s13195-017-0248-8 (PMC5361707; doi:10.1186/s13195-017-0248-8)
Supplement: Supplementary file 1 — is a figure showing the experimental flow chart. (DOCX 37 kb) [file 13195_2017_248_MOESM1_ESM.docx]

**Additional file 1**

**a**

**b**

**c**

**Additional file 1. Clinical and pathological states of the study cohort** (a) Standardized uptake value ratio (SUVR) of subjects. (*P < 0.05 and ***P < 0.001, analyses of variance (ANOVA) followed by Tukey’s multiple comparison test). (b) Mini-mental state examination (MMSE) z-score (*P < 0.05, ANOVA followed by Tukey’s multiple comparison test). (c) Clinical dementia rating (CDR) score (***P < 0.001, ANOVA followed by Tukey’s multiple comparison test). - or +, PiB-PET positivity; CN, cognitively normal subjects; MCI, subjects with mild cognitive impairment; ADD+, patients with Alzheimer’s disease dementia with amyloid deposition; ADD-, patients with Alzheimer’s disease dementia with negative amyloid deposition; ; MMSE z-score, a revised value of the MMSE score with consideration for age, gender, and education level.
