# Supplementary material for: Chemically treated plasma Aβ is a potential blood-based biomarker for screening cerebral amyloid deposition
Source: Alzheimers Res Ther. 2017 Mar 22;9:20. doi: 10.1186/s13195-017-0248-8 (PMC5361707; doi:10.1186/s13195-017-0248-8)
Supplement: Supplementary file 2 — is a table presenting demographic data of the complete study cohort. (DOCX 58 kb) [file 13195_2017_248_MOESM2_ESM.docx]

**Additional file 2**

**a**

**b**

**Additional file 2. MPP-Aβs and aging** (a) CN- show significantly higher MPP-Aβ42 and MPP-Aβ42/40 ratio than YC (***P < 0.001, unpaired *t*-test). (b) MPP-Aβ42 and MPP-Aβ42/40 ratio show significant association with aging (***P < 0.0001, r = 0.40 for MPP-Aβ42; ***P < 0.0001, r = 0.33 for MPP-Aβ42/40 ratio; Pearson’s correlation). MPP, Mixture of Protease inhibitors and Phosphatase inhibitors; YC, Young-middle-aged controls (cognitively normal subjects with age 20 to 55 years, n = 61); CN-, cognitively normal PiB-PET negative subjects (n = 187).
