# Supplementary material for: Chemically treated plasma Aβ is a potential blood-based biomarker for screening cerebral amyloid deposition
Source: Alzheimers Res Ther. 2017 Mar 22;9:20. doi: 10.1186/s13195-017-0248-8 (PMC5361707; doi:10.1186/s13195-017-0248-8)
Supplement: Supplementary file 3 — is a figure showing clinical and pathological states of the study cohort: (a) SUVR of subjects (*P < 0.05 and ***P < 0.001, ANOVA followed by Tukey’s multiple comparison test), (b) MMSE z score (*P < 0.05, ANOVA followed by Tukey’s multiple comparison test), and (c) CDR score (***P < 0.001, ANOVA followed by Tukey’s multiple comparison test). (DOCX 67 kb) [file 13195_2017_248_MOESM3_ESM.docx]

**Additional file 3**

**
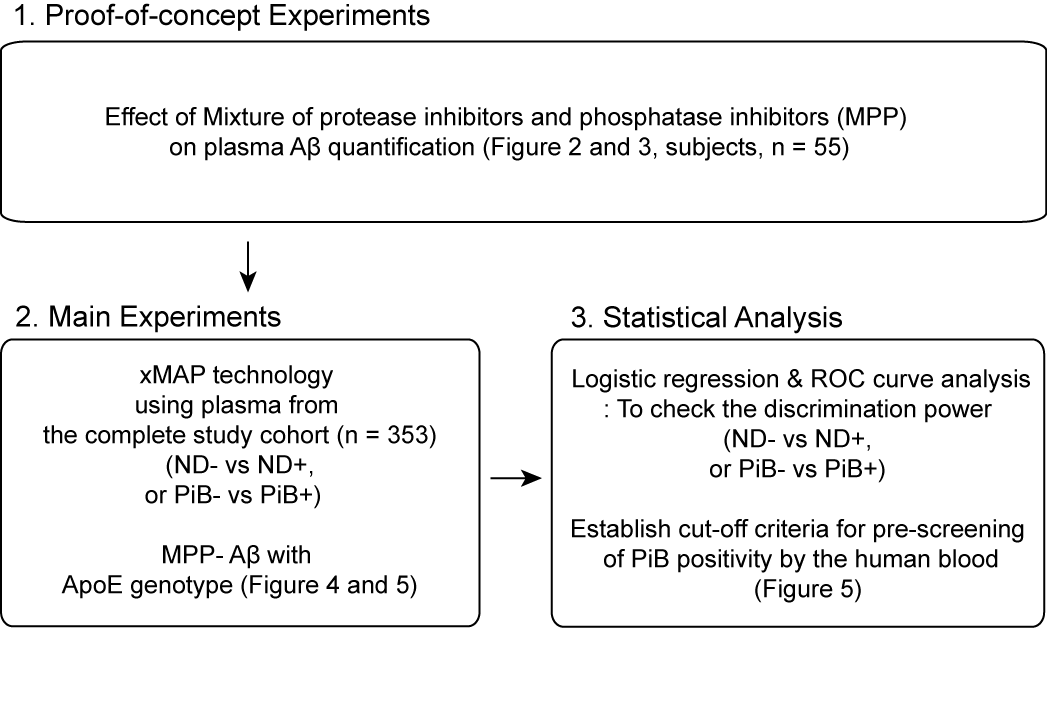
Additional file 3. Experimental flow chart.** MPP, mixture of protease inhibitors and phosphatase inhibitors; ROC curve, receiver operating characteristic curve; ApoE, Apolipoprotein E; ND-, non-demented PiB-PET negative subjects, CN- and MCI-; ND+, non-demented PiB-PET positive subjects, CN+ and MCI+; CN-, MCI-, and ADD- were grouped as PiB-; CN+, MCI+, and ADD+ were grouped as PiB+; CN, cognitively normal subjects; MCI, subjects with mild cognitive impairment; ADD, patients with Alzheimer’s disease dementia.
